# Function of the Deubiquitinating Enzyme USP46 in the Nervous System and Its Regulation by WD40-Repeat Proteins

**DOI:** 10.3389/fnsyn.2017.00016

**Published:** 2017-12-14

**Authors:** Molly Hodul, Caroline L. Dahlberg, Peter Juo

**Affiliations:** ^1^Department of Developmental, Molecular and Chemical Biology, Tufts University School of Medicine, Tufts University, Boston, MA, United States; ^2^Graduate Program in Neuroscience, Sackler School of Graduate Biomedical Sciences, Tufts University School of Medicine, Tufts University, Boston, MA, United States; ^3^Biology Department, Western Washington University, Bellingham, WA, United States

**Keywords:** ubiquitin, deubiquitinating enzymes, USP46, AMPA receptors, WD40 repeat protein, WDR48, WDR20, USP12

## Abstract

Posttranslational modification of proteins by ubiquitin regulates synapse development and synaptic transmission. Much progress has been made investigating the role of ubiquitin ligases at the synapse, however very little is known about the deubiquitinating enzymes (DUBs) which remove ubiquitin from target proteins. Although there are far fewer DUBs than ubiquitin ligases encoded by the human genome, it is becoming clear that DUBs have very specific physiological functions, suggesting that DUB activity is tightly regulated *in vivo*. Many DUBs function as part of larger protein complexes, and multiple regulatory mechanisms exist to control the expression, localization and catalytic activity of DUBs. In this review article, we focus on the role of the DUB USP46 in the nervous system, and illustrate potential mechanisms of regulating DUBs by describing how USP46 is regulated by two WD40-repeat (WDR) proteins, WDR48/UAF1 and WDR20, based on recent structural studies and genetic analyses *in vivo*.

## Introduction

Ubiquitination is a widely used posttranslational modification that has emerged as a key regulator of synapse development and function (DiAntonio and Hicke, 2004; Mabb and Ehlers, 2010). Ubiquitination of a growing list of pre- and postsynaptic proteins can regulate their stability, function and subcellular localization (Mabb and Ehlers, 2010; Bingol and Sheng, 2011; Kowalski and Juo, 2012). The covalent attachment of ubiquitin to lysine residues on target proteins is catalyzed by a sequence of enzymatic reactions mediated by E1 ubiquitin-activating enzymes, E2 ubiquitin-conjugating enzymes and E3 ubiquitin ligases (Hershko and Ciechanover, 1998). Ubiquitin itself has seven lysine residues and a primary amine at its N-terminus that can be utilized to form ubiquitin chains. Monoubiquitination and polyubiquitination, using various chain-linkage types, create distinct structural topologies that are recognized by ubiquitin binding proteins that mediate the various functions of ubiquitin. For example, Lys48-and Lys11-linked polyubiquitination result in proteasomal degradation, whereas Lys63-linked polyubiquitination is typically associated with signaling and endo-lysosomal trafficking (Piper and Lehner, 2011; Clague et al., 2012; Nathan et al., 2013). Ubiquitin can be removed from substrates by proteases called deubiquitinating enzymes (DUBs). The human genome encodes approximately 100 DUBs that are categorized into six families. The ubiquitin-specific proteases (USPs) make up the largest family, comprised of 54 cysteine proteases (Nijman et al., 2005; Mevissen and Komander, 2017).

There are six times more ubiquitin ligases than DUBs in the human genome, suggesting that DUBs might have more promiscuous substrate specificity than ubiquitin ligases (Komander et al., 2009). However, emerging evidence indicate that DUBs have very specific cellular functions and are selective for certain substrates and ubiquitin chain types, suggesting that precise mechanisms exist to regulate DUBs (Clague et al., 2013). Although some DUBs, such as UCH-L1, have been heavily studied in the nervous system, the function and regulation of most neuronal DUBs are poorly understood (Todi and Paulson, 2011; Kowalski and Juo, 2012). In this review article, we illustrate potential mechanisms of DUB regulation by focusing on the role of the conserved DUB USP46 in the nervous system, and ways in which two WD40-repeat (WDR) proteins control its function. We highlight recent structural insights into how WDR proteins interact with and regulate USP46, and discuss functions for the USP46/WDR protein complex in the nervous system and across phylogeny.

## USP46 Regulates Glutamate Receptors in *C. elegans* and Mammals

AMPA-type glutamate receptors (AMPARs) mediate the majority of fast excitatory transmission in the brain, and regulation of synaptic AMPAR levels is important for controlling synapse development and function. AMPARs are assembled as hetero-tetramers comprised of various combinations of the pore-forming subunits GluA1-A4. Subunit composition and association with auxiliary subunits determine the biophysical and trafficking properties of the channel (Anggono and Huganir, 2012). AMPARs can be regulated by multiple posttranslational modifications, such as ubiquitination, which controls receptor trafficking and degradation (Goo et al., 2015). Studies of the AMPAR GLR-1, which shares 40%–50% identity with rat GluA1 and GluA2 (Hart et al., 1995; Brockie et al., 2001), in *C. elegans* were the first to show that glutamate receptors (GluRs) are regulated by ubiquitin (Burbea et al., 2002). Ubiquitin is directly conjugated to the cytoplasmic tail of GLR-1, providing a signal for clathrin-mediated endocytosis and subsequent degradation (Burbea et al., 2002). Later studies showed that all four mammalian AMPAR subunits, GluA1-A4, are also regulated by ubiquitin (Schwarz et al., 2010; Fu et al., 2011; Lin et al., 2011; Lussier et al., 2011; Widagdo et al., 2015). Although several studies suggest that AMPARs are likely ubiquitinated at the plasma membrane prior to internalization (Burbea et al., 2002; Schwarz et al., 2010; Lin et al., 2011), other studies propose that AMPARs are ubiquitinated at endosomes (Lussier et al., 2011; Widagdo et al., 2015), prior to degradation in the lysosome. Future research will be necessary to determine if stimulus type, intensity or duration determine the subcellular site of ubiquitination.

While several ubiquitin ligases have been shown to regulate AMPARs (reviewed in Goo et al., 2015), much less is known about the relevant DUBs. USP-46 was identified in a focused RNAi screen in *C. elegans* as the first DUB to regulate GluRs. *Usp-46* loss-of-function mutants exhibit increased levels of ubiquitinated GLR-1 and decreased levels of GLR-1 at synapses (Kowalski et al., 2011). The abundance of GLR-1(4KR), a mutant receptor which cannot be ubiquitinated, is unaffected in *usp-46* loss-of-function mutants leading to a model where USP-46 deubiquitinates GLR-1 to regulate its abundance. USP-46 regulation of GLR-1 is physiologically relevant because *usp-46* loss-of-function mutants have reduced cell surface levels of GLR-1 and corresponding defects in GLR-1-dependent behaviors. Together with data showing that USP-46 partially colocalizes with endosomes, Kowalski et al. (2011) proposed a model where USP-46 acts at endosomes to promote GLR-1 stability and recycling to the cell surface. Consistent with this model, a recent study showed that mammalian GluA1(KR), which cannot be ubiquitinated, escapes lysosomal degradation and recycles back to the cell surface (Widagdo et al., 2015).

*C. elegans* USP-46 is highly homologous to both mammalian USP46 and its paralog USP12 (the paralogs share 88% amino acid identity; Kowalski et al., 2011). A recent study using cultured rodent neurons showed that USP46 regulation of AMPARs is conserved in mammals. Mammalian USP46 can deubiquitinate both GluA1 and GluA2 subunits and protect AMPARs from degradation (Huo et al., 2015). USP46 is expressed throughout the brain, including the hippocampus, amygdala, cerebellum and prefrontal cortex, and colocalizes with GluA1 and PSD95 at synapses in cultured neurons (Tomida et al., 2009; Huo et al., 2015). Knock-down of USP46, but not USP12, results in increased levels of ubiquitinated GluA1, decreased surface and total levels of GluA1, and reduced mEPSC amplitudes, consistent with a role for USP46 in deubiquitinating mammalian AMPARs (Huo et al., 2015). Interestingly, GluA1 is preferentially modified with Lys63-linked polyubiquitin chains, which typically promotes endo-lysosomal trafficking (Huo et al., 2015; Widagdo et al., 2015). Prior studies showed that USP enzymes have promiscuous chain selectivity and recombinant USP46 prefers Lys6- and Lys11-linked polyubiquitin chains *in vitro* (Faesen et al., 2011). In contrast, Huo et al. (2015) showed that USP46 preferentially deubiquitinates AMPARs with Lys63- but not Lys48-linked chains in HEK293 cells, suggesting that USP46 chain specificity is controlled by other factors *in vivo*. Together, these data reveal a conserved mechanism where USP46 deubiquitinates AMPARs at synapses to protect them from degradation and promote their recycling to the cell surface to affect synapse function. In addition to USP46, USP8 can also deubiquitinate mammalian AMPARs indicating that multiple regulatory mechanisms exist to control AMPAR ubiquitination levels (Scudder et al., 2014).

## Effects of USP46 on the GABA System

Ionotropic GABA_A_ receptors mediate the majority of fast inhibitory transmission in the brain. GABA_A_ receptors are comprised of hetero-pentamers and ubiquitination of specific subunits can regulate receptor trafficking and degradation in an activity-dependent manner (Saliba et al., 2007; Jacob et al., 2008; Arancibia-Cárcamo et al., 2009).

USP46 is also implicated in regulating the GABAergic system in mice. Tomida et al. (2009) discovered that an inbred strain of mice (CS strain) known to have defects in circadian rhythms, also exhibits changes in depression-like behaviors consistent with an anti-depressive state. Quantitative trait locus mapping of the CS mice identified a 3bp deletion in a conserved lysine (ΔK92) in USP46. Importantly, USP46 knock-out (KO) mice exhibit similar changes in depression-like behaviors (Imai et al., 2013) and broad expression of a wild type USP46 transgene in CS mice rescued these behaviors (Tomida et al., 2009). The magnitude of behavioral effects in the USP46 (ΔK92) mutant mice were not as strong as those observed in USP46 KO mice (Imai et al., 2013), suggesting that the ΔK92 mutation does not completely eliminate USP46 activity. Indeed, *in vitro* deubiquitination assays revealed that USP46(ΔK92) still retains some enzymatic activity (Zhang et al., 2011).

Several experiments suggest that USP46 loss-of-function affects the GABAergic system. First, hippocampal immunohistochemistry showed reduced expression of the GABA synthesis enzyme glutamic acid decarboxylase (GAD67) in USP46 (ΔK92) mutants (Tomida et al., 2009), suggesting that USP46 may regulate GABA synthesis. Second, muscimol-induced postsynaptic GABA_A_ receptor currents were slightly reduced in hippocampal neurons of USP46 (ΔK92) mutants. This effect may be mediated by extrasynaptic GABA_A_ receptors since no alterations were observed in mIPSC amplitude or frequency (Tomida et al., 2009). Third, administration of the GABA_A_ receptor agonist, nitrazepam, restores depression-like behavior in USP46 (ΔK92) and KO mutant mice, and these effects could be blocked by the GABA_A_ receptor antagonist flumazenil (Imai et al., 2012). Together, these data suggest that USP46 affects both pre- and postsynaptic components of the GABA system, however the precise mechanism is not known. One possibility is that USP46 may indirectly affect the GABA system as a compensatory response to a primary defect in AMPAR degradation in excitatory neurons. It will be important in the future to test if USP46 functions in GABA neurons and whether it directly deubiquitinates components of the GABA system.

## Regulation by WD40-Repeat Proteins

WDR proteins are involved in protein-protein interactions that mediate diverse cellular processes. WDRs consist of 40–60 amino acids ending in a tryptophan-aspartic acid (WD) motif. The WDR form a funnel-shaped, β-propeller structure made up of 6–8 blades, with each blade consisting of four anti-parallel β-sheets that are held together by extensive hydrogen bonds. This rigid β-propeller structure provides multiple stable surfaces for protein interactions (Pashkova et al., 2010; Stirnimann et al., 2010; Villamil et al., 2013).

Mammalian USP46 and USP12 are comprised largely of a core catalytic domain that exhibits low intrinsic activity (Cohn et al., 2009; Kee et al., 2010; Faesen et al., 2011). Biochemical and proteomic studies showed that two WDR proteins, WDR48 (also known as USP1-associated factor, UAF1) and WDR20, interact with USP46 and USP12 (Cohn et al., 2009; Sowa et al., 2009; Kee et al., 2010). WDR48 stimulates the activity of three DUBs, USP12, USP46 and USP1, a DUB which regulates the Fanconi anemia DNA damage pathway (Cohn et al., 2007, 2009; Faesen et al., 2011). WDR20 forms a unique ternary complex with WDR48 and either USP12 or USP46 (Sowa et al., 2009; Kee et al., 2010) and further enhances their catalytic activity *in vitro* (Kee et al., 2010; Faesen et al., 2011). Intriguingly, a large-scale proteomic analysis of DUB-interacting proteins in HEK293 cells revealed that 36% of the 75 DUBs analyzed interact with a WDR protein, suggesting a broad role for WDR proteins in regulating DUBs (Sowa et al., 2009).

**Figure 1:**
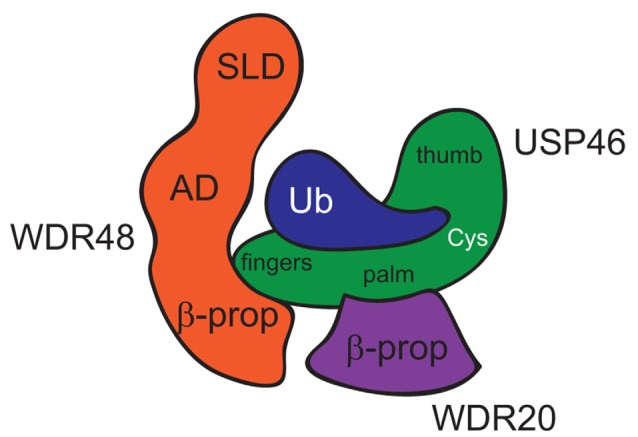
Model of USP46 bound to WDR48 and WDR20. This model is inferred from a combination of structures of USP46 bound to WDR48 and ubiquitin (Yin et al., 2015) and USP12 bound to WDR48 and WDR20 (Li et al., 2016). SLD, Sumo-Like Domain; AD, Ancillary Domain; β-prop, β-propellar of WD40-repeat domain; Cys, active-site Cysteine residue.

Both WDR48 and WDR20 stimulate USP12 and USP46 catalytic activity (k_cat_) without increasing substrate binding affinity (K_m_; Faesen et al., 2011; Dharadhar et al., 2016; Li et al., 2016), suggesting that the WDR proteins may affect DUB activity via a novel structural mechanism. Three recent studies provide crystal structures of the WDR proteins in complex with USP12 and USP46. USP12 and USP46 were each crystallized bound to WDR48/UAF1 (Yin et al., 2015; Dharadhar et al., 2016; Li et al., 2016), and USP12 was also crystalized in a ternary complex with WDR48 and WDR20 (Li et al., 2016). WDR48 and WDR20 bind the DUBs relatively far from the catalytic cleft and stimulate DUB catalytic activity via allosteric mechanisms (Figure 1; Yin et al., 2015; Dharadhar et al., 2016; Li et al., 2016). USP12 and USP46 exhibit the conserved USP fold structure comprised of Fingers, Palm and Thumb subdomains, with the catalytic triad of cysteine, histidine and aspartic acid, nestled in between the Palm and Thumb regions. The “top” narrow end of the β-propeller funnel of WDR48 interacts with the tip of the USP Fingers subdomain, whereas the WDR48 ancillary domain (AD) and sumo-like domain (SLD) curve around ubiquitin, which binds in the Palm region of the DUB. The C-terminal glycine residue and tail of ubiquitin extend towards the active site cysteine of the DUB. While Yin et al. (2015) and Dharadhar et al. (2016) crystallized their complexes in the presence of an ubiquitin-bound substrate, Li et al. (2016) crystallized USP12 in complex with WDR48 and WDR20 in the absence of bound-ubiquitin, providing additional structural insight. For example, the “Pinky Finger” β-sheet of the Fingers subdomain appears disordered or displaced in apoUSP12, whereas the four antiparallel β-sheets of the Fingers subdomain are rigid in other USP structures (Hu et al., 2002; Avvakumov et al., 2006; Ratia et al., 2006; Renatus et al., 2006; Yin et al., 2015). Binding of WDR48 to the tip of the Fingers subdomain stabilizes the “Pinky Finger” and Fingers subdomain, which may ultimately propagate to the catalytic cleft in the presence of substrate (Li et al., 2016). WDR20 also binds the DUB via the “top” face of its β-propeller but interacts with the bottom of the palm subdomain of USP12 to promote an optimal alignment of the catalytic cleft (Li et al., 2016). Together, these structural studies suggest that binding of the WDR proteins to USP12 or USP46 relatively far from the active site results in the rearrangement of several structural elements, which propagates to the catalytic triad increasing enzyme catalysis.

These recent structural and biochemical studies complement *in vivo* studies of the USP-46/WDR-48/WDR-20 complex in *C. elegans*. Dahlberg and Juo (2014) showed that the* C. elegans* homologs of WDR48 and WDR20 form a stable ternary complex with USP-46 in HEK293T cells. Consistent with prior studies (Cohn et al., 2009; Kee et al., 2010; Faesen et al., 2011), full activation of *C. elegans* USP-46 catalytic activity *in vitro* requires both WDR48 and WDR20 (Dahlberg and Juo, 2014). In contrast to the increased degradation of GLR-1 observed in *usp-46* loss-of-function mutants (Kowalski et al., 2011), overexpression of *usp-46* in neurons does not stabilize GLR-1 (Dahlberg and Juo, 2014). However, co-expression of WDR-48 and WDR-20 in neurons did increase surface and total levels of GLR-1 as well as glutamate-dependent behavior in an *usp-46*-dependent manner. These data suggest that endogenous WDR-48 and WDR-20 may be limiting *in vivo*. Together with data showing that co-expression of the USP-46/WDR-48/WDR-20 complex reduces levels of ubiquitinated GLR-1 (Dahlberg and Juo, 2014), these results support a model wherein USP-46, when bound to WDR-48 and WDR-20, deubiquitinates GLR-1 and increases receptor stability and function *in vivo*.

Interestingly, Huo et al. (2015) showed that knock-down of mammalian USP46 has a greater effect on reducing surface GluA1 (42% decrease) and mEPSC amplitude (32% decrease) than overexpression of USP46 (18%–20% increase in surface GluA1 and mEPSC amplitudes; Huo et al., 2015). The more modest effects of USP46 overexpression are consistent with the low intrinsic catalytic activity of USP46 in the absence of WDR48 and WDR20 (Cohn et al., 2009; Faesen et al., 2011). Intriguingly, Huo et al. (2015) also showed that mammalian USP46, but not USP12, was able to regulate GluA1 in neurons. It will be interesting to determine how USP46 and USP12 achieve substrate specificity *in vivo* given that they can both interact with WDR48 and WDR20.

## Phylogenetic Conservation of WD40-Repeat Proteins and USP46

WDR proteins and USP46 are conserved across phylogeny from yeast to humans (Table 1). Studies in the multicellular filamentous fungi *Aspergillus nidulans* were the first to describe a role for WDR proteins in regulating DUBs (Lockington and Kelly, 2001, 2002). CreB, which is homologous to USP46 and USP12, and CreC, which is homologous to WDR20, were identified in a screen for genes involved in carbon catabolite repression and gene expression. Genetic experiments revealed that CreC acts upstream of CreB and that CreC stabilizes CreB by interacting with and preventing proteolysis of the DUB (Lockington and Kelly, 2002).

**Table 1:** Homologs of USP46, WDR48 and WDR20 across phylogeny.

Species	USP46/USP12 homologs	WDR48 homologs	WDR20 homologs
*H. sapiens*^1–3^	USP46, USP12	WDR48/UAF1	WDR20
*M. musculus*^1–3^	USP46, USP12	WDR48/UAF1	WDR20
*D. melonagaster*^4,5^	CG7023/USP12	CG9062	CG6420
*C. elegans*^6,7^	USP-46	WDR-48	WDR-20
*A. nidulans*^8,9^	CreB	?	CreC
*S. cerevisiae*^10^	Ubp9, Ubp13	Duf1	?
*S. pombe*^11^	Ubp9	Bun107	Bun62

*References: ^*1*^Cohn et al. (2009); ^*2*^Sowa et al. (2009); ^*3*^Kee et al. (2010); ^*4*^Moretti et al. (2012); ^*5*^Tsou et al. (2012); ^*6*^Kowalski et al. (2011); ^*7*^Dahlberg and Juo (2014); ^*8,9*^Lockington and Kelly (2001, 2002); ^*10*^Kanga et al. (2012); ^*11*^Kouranti et al. (2010)*.

In the unicellular fission yeast *S. pombe*, the USP46 homolog Ubp9 regulates endocytosis, actin dynamics and cell polarity (Kouranti et al., 2010). Much like its counterparts in other systems, Ubp9 is only catalytically active when bound to two WDR proteins, Bun107, which is homologous to WDR48, and Bun62, which is homologous to WDR20. Interestingly, Ubp9 stability and subcellular localization can be regulated by interaction with the WDR proteins.

In the budding yeast *S. cerevisiae*, the USP46 homolog Ubp9, and the closely related DUB Ubp13, regulate mitochondrial function by controlling the biosynthesis of a key ATP synthase subunit (Kanga et al., 2012). These DUBs interact with the WDR48 homolog Duf1. Consistent with other studies, Duf1 stimulates Ubp9 and Ubp13 catalytic activity and is required for the physiological function of these enzymes *in vivo*. Interestingly, the WD40 domain of Duf1 can interact directly with ubiquitin (Pashkova et al., 2010), hinting at another potential role for these WDR proteins.

Similar to *C. elegans*, the genome of the fruit fly *Drosophila melonagaster* encodes for one DUB, CG7023, that is homologous to both USP46 and USP12 (Moretti et al., 2012; Tsou et al., 2012) and one homolog each of WDR48 and WDR20 (Table 1). CG7023/USP12 was shown to negatively regulate Notch signaling in flies (Moretti et al., 2012). Partial RNAi knock-down of CG7023/USP12 in the fly nervous system did not reveal any obvious neuronal phenotypes (Tsou et al., 2012). It will be informative to investigate the null phenotype of the WDR proteins and USP12 in the fly nervous system.

## Concluding Remarks

DUBs have emerged as critical regulators of a large number of ubiquitin-dependent processes including synapse development and function. Growing evidence indicates that DUB localization and activity are tightly controlled *in vivo* through protein-protein interactions. Here, we highlighted recent progress in our understanding of how WDR proteins, WDR48 and WDR20, interact with and activate USP12 and USP46. Future studies should reveal if WDR48 and WDR20 expression or subcellular localization are regulated in neurons as a mechanism to control DUB function. Understanding how DUB function is regulated *in vivo* will provide critical information for the design of better drugs to fine-tune ubiquitin-regulation of protein trafficking or degradation of key proteins. Interestingly, although one human genetic study found no association between USP46 mutations and bipolar disorder or schizophrenia (Kushima et al., 2010), another study identified USP46 as a candidate gene associated with early-onset essential tremor (Liu et al., 2016). It will be interesting to learn if mutations in USP46 or its regulators are associated with other neurological disorders given the importance of USP46 in both glutamatergic and GABAergic signaling.

## Author Contributions

MH, CLD and PJ wrote the manuscript and contributed to the Figure and Table.

## Conflict of Interest Statement

The authors declare that the research was conducted in the absence of any commercial or financial relationships that could be construed as a potential conflict of interest.
